# Ophthalmologic Findings in Children with Leukemia: A Single-Center Study

**DOI:** 10.4274/tjo.03880

**Published:** 2016-04-05

**Authors:** Betül Orhan, Barış Malbora, Sezin Akça Bayar, Zekai Avcı, Bülent Alioğlu, Namık Özbek

**Affiliations:** 1 Başkent University Faculty of Medicine, Department of Pediatrics, Ankara, Turkey; 2 Başkent University Faculty of Medicine, Department of Pediatric Hematology, Ankara, Turkey; 3 Başkent University Faculty of Medicine, Department of Ophthalmology, Ankara, Turkey

**Keywords:** children, Leukemia, ophthalmologic findings

## Abstract

**Objectives:**

Ophthalmologic disease in patients with acute leukemia occurs due to primary leukemic infiltration (involvement), or secondary to the disease and its treatment. In recent years the life expectancy of acute leukemia patients has increased with the advent of modern therapies. The present study aimed to determine the incidence of ocular manifestations in children with acute leukemia.

**Materials and Methods:**

The study included 120 patients diagnosed with acute leukemia at Başkent University Hospital, Pediatric Hematology Department between 1995 and 2010. All the patients were examined by an ophthalmologist via direct and indirect ophthalmoscopy.

**Results:**

Among the patients, 83 (69.2%) were diagnosed with acute lymphoblastic leukemia, 35 (29.1%) with acute myeloblastic leukemia, and 2 (1.7%) with mixed-lineage leukemia. In all, 58 ophthalmic manifestations were noted in 41 patients (34.2%). In our patients, 12 ophthalmologic involvements were present at admission and 46 ocular findings occurred during follow-up. The incidence of these manifestations increased with age.

**Conclusion:**

Ophthalmologic manifestations were not correlated with gender, hematological parameters at disease onset, type of leukemia, or the frequency of relapse and survival. To more clearly determine the effect of ophthalmologic manifestations on the prognosis of leukemia, larger scale and multi-center studies are needed.

## INTRODUCTION

Though less commonly observed compared to the other organs, ocular findings may occur in patients with leukemia.^1^ Since the initial description of leukemic retinopathy in the 1860s, it has been shown that nearly all eye structures may be affected in leukemia patients.^2^ Since the mean life expectancy of leukemic patients is increasing due to the advances in diagnosis and treatment, the incidence of ocular findings is increasing. Ophthalmic signs in leukemia can be observed at the onset of disease or during follow-up. According to the literature, ocular involvement occurs in 9% to 90% of leukemia patients and most frequently affects the retina;^3,4^ however, the relationship between the prognosis of leukemia and ocular manifestations remains unclear. In the present study, we aimed to investigate ophthalmic manifestations in patients with acute leukemia and to determine whether there is a relationship between these manifestations and morbidity or prognosis.

## MATERIALS AND METHODS

The study included 120 children with acute leukemia who were treated in our department between 1995 and 2010. Patient age, gender, hematologic parameters at diagnosis, organomegaly, and extramedullary involvement was recorded.

Acute leukemias were classified according to the French-American-British (FAB) classification as follows: acute lymphoblastic leukemia (ALL), acute myeloblastic leukemia (AML), and acute mixed leukemia. For morphological classification, a bone marrow aspirate was stained with Wright stain, then examined with an optical microscope. Immunophenotyping was performed via flow-cytometry (Becton Dickons Canto II, Sysmex XT-2000i®). Conventional cytogenetic analysis was performed in all patients. Since 2003, fluorescence in situ hybridization analysis for specific areas of chromosomes was performed, as well as chromosomal analysis. Patients diagnosed with ALL between 1995 and 2005 were given the St. Jude Total XIII protocol, those diagnosed with ALL between 2005 and 2006 were given the ALL-Berlin-Frankfurt-Munster-1990 (ALL-BFM 90) protocol, and those diagnosed with ALL between 2006 and 2010 received the ALL-BFM 95 protocol. Patients diagnosed with AML received the AML-Berlin-Frankfurt-Munster-1993 (AML-BFM 93) protocol between 1995 and 2007, and after 2007 they received the AML-BFM 2004 protocol.

Findings at admission (FAA) were defined as the ocular manifestation(s) observed at the time of leukemia diagnosis, whereas findings during follow-up (FDF) were defined as ocular manifestations observed during or after the treatment of leukemia. At first admission with the diagnosis of acute leukemia, the patients underwent a detailed ophthalmologic examination by an ophthalmologist. All the patients who were followed in our department were also examined at remission and at the end of treatment. Ophthalmologic examinations were repeated in cases of relapse or any complaints concerning the eyes. Ocular manifestations at the time of leukemia diagnosis and during the course of follow-up were recorded. Visual acuity measurement and biomicroscopic examination including direct and indirect ophthalmoscopy were performed in all patients at diagnosis and during follow-up. Fundus photographs were obtained from selected patients as needed.

### Statistical Methods

Statistical evaluation of the data was performed using Statistical Package for the Social Sciences v.19.0 for Windows. Benchmark analysis of categorical data was performed using chi-square and Fisher’s exact tests, Kruskal-Wallis ANOVA analysis of quantitative data and Mann-Whitney U test were used. Arithmetic mean ± standard deviation and median values were used for quantitative data and frequency and percentage were used for qualitative data as descriptive statistics. The level of statistical significance was set at α=0.05.

The present study was performed in accordance with the ethical standards set forth in the 1964 version of the Declaration of Helsinki, and the Başkent University Ethics Committee approved the study protocol.

## RESULTS

In total, 83 (69.2%) patients had ALL, 35 (29.1%) had AML, and 2 (1.7%) had acute mixed leukemia. Table 1 shows the patients’ demographic data. The distribution of gender, leukemic cell morphology, and immune-phenotype did not differ between the patients with ALL and AML. Considering complete blood count data at diagnosis, only the platelet counts were different, being lower in AML patients compared to those in patients with ALL (p=0.002). In all, 30% of the AML patients and 33% of ALL patients admitted with a leukocyte count >20x109/L. However, there was no correlation between the high leukocyte counts and ophthalmologic manifestations in our patients.

### Extramedullary Involvement

In total, 39 (32.5%) of the patients had extramedullary involvement at the time of diagnosis; 64% (n=25) of cases were observed in ALL patients, versus 36% (n=14) in AML patients (Table 1). The most frequently involved extramedullary organ was the kidney [n=12 (31%)], followed by the central nervous system (CNS) [n=9 (23%)], gastrointestinal system [n=6 (15.4%)], eye [n=5 (13%)], bone [n=2 (5.1%)], pleura [n=2 (5%)], pericardium [n=2 (5%)] and thymus [n=1 (2.5%)]. Eye and gastrointestinal involvement were more common in the AML patients than in the ALL patients, but the difference was not significant (p=0.053) (Table 1). Relapse occurred in 20 of the patients with acute leukemia. Only one of the patients with acute mixed-lineage leukemia had orbital relapse together with CNS involvement 10 months after diagnosis. He was given a protocol for the treatment of children with relapsed ALL (ALL REZ-BFM relapse protocol) and cranio-spinal radiotherapy was administered. The last eye examination was normal. The patient later died due to sepsis.

### Ophthalmologic Manifestations and Anatomic Distribution

Ophthalmologic manifestations occurred in 41 (34.2%) of the patients (17 male and 24 female) with acute leukemia. Among these, 32 had manifestations at the time of diagnosis (FAA), whereas 9 of them developed secondary ophthalmic manifestations after the diagnosis (FDF) (mean 9 months). There were no significant differences in ophthalmic manifestations according to the patients’ leukemia morphology or immune-phenotype. Since some patients had multiple ophthalmic manifestations, 58 ophthalmic manifestations in total were observed in 41 patients. In our study, survival and relapse rates were similar in children who had ophthalmological manifestations at admission compared to those without ophthalmological findings.

Mean age at diagnosis of leukemia was higher in the patients with ophthalmologic manifestations (for ALL 6.4, AML 10.9, total 7.9 years) than those without ophthalmologic involvement (for ALL 5.2, AML 7.6, total 5.9 years). This difference was significant in patients with AML (n=35, p=0,006) and it was consistent when we analyzed all the patients with leukemia (n=120, p=0.04). Although the mean age at diagnosis was also higher in ALL patients with ophthalmologic findings, the difference was not significant (n=83, p=0.375).

**Figure 1:**
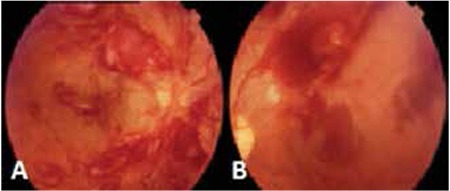
The retinal hemorrhages of patients with acute leukemia (A, right eye; B, left eye)

In all, ophthalmic manifestations observed in our patients were mostly FDF [46 FDF manifestations (79%) vs 12 FAA manifestations (21%)]. The retina was the most common site of involvement. In total, there were 16 retinal manifestations (2 FAA and 14 FDF). Retinal manifestations did not differ significantly according to the hematological parameters. Among patients with retinal involvement at admission, an 18-month-old male diagnosed with B-cell ALL and significant leukocytosis (400x109/L) at the time of diagnosis showed extended leukemic mass below the retina and retinal detachment (Figure 1). Following leukapheresis and systemic chemotherapy, the mass disappeared. Unfortunately, approximately 1 year after diagnosis, the patient had bone marrow relapse without ocular involvement. He underwent bone marrow transplantation, but had another relapse after transplantation and passed away. The other patient with retinal involvement at admission was a 14-year-old female diagnosed with secondary AML-M5 who had completed chemotherapy for osteosarcoma 2 years earlier. At the initial examination, the patient had blurred vision and serous retinal detachment. An orbital tumor was also observed via cranial magnetic resonance imaging. Cytogenetic analysis of the bone marrow was normal. Following treatment, her ocular symptoms improved; however, during the third month of treatment she died due to sepsis. Another patient with retinal detachment at diagnosis was a 15-year-old male with AML-M2 blast cell morphology and t(8;21) translocation. His hemoglobin level was 5.1 g/dl; leukocyte and thrombocyte counts were 6,100/mm3 and 7,000/mm3 respectively. In addition to the retinal detachment, intraretinal hemorrhage and orbital granulocytic sarcoma were observed at time of diagnosis. The patient was treated with AML BFM 93 protocol and short-term high dose methylprednisolone. His ocular findings completely resolved after the methylprednisolone treatment.^5^

**Figure 2:**
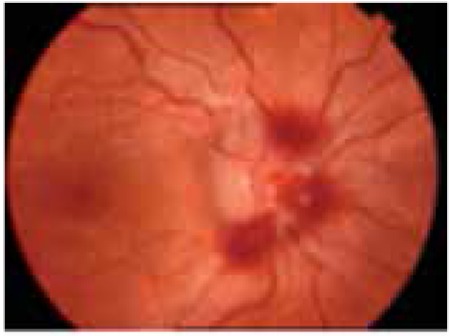
Retinal detachment in a patient with acute leukemia

**Figure 3:**
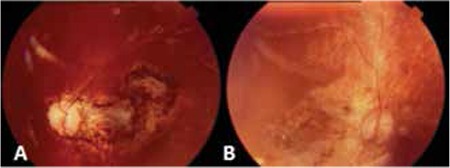
Retinal scar in a patient with acute myeloblastic leukemia (A, right eye; B, left eye)

Retinal pathologies during follow-up included retinal hemorrhage (n=10; 8 in ALL and 2 in AML patients), retinal detachment (n=2; 1 ALL and 1 AML), and retinitis (n=2; both AML) in our patients (Figure 2). Retinitis was noted in 2 patients with AML. One of them was diagnosed with FAB AML-M5 at the age of 7 months. During maintenance of AML-BFM treatment, he developed cytomegalovirus (CMV) retinitis treated with intravenous ganciclovir, then with oral valganciclovir for 1 year. This patient healed with a retinal scar and as of the time this manuscript was prepared had been in remission for 3 years (Figure 3).

Conjunctival manifestations were observed in 15 patients (10 ALL and 5 AML). Conjunctival involvement at admission was observed in only 1 patient, a 12-year-old female with pre-B-cell ALL. Leukemic infiltration was present in the right auricle along with both conjunctivas. Leukemic infiltration was noted in samples obtained from the ear, but conjunctival samples could not be obtained. The patient started chemotherapy; her ophthalmologic evaluation was normal after two weeks of treatment. She has been in remission for the last 7 years. In all, 14 patients had conjunctival manifestations during follow-up, of which 9 (6 ALL and 3 AML) had conjunctivitis. In 8 of these patients, conjunctivitis was infectious in origin and in the other it was allergic. In 2 of the 5 patients with conjunctival hemorrhage the platelet count was <20x109/L at the time of hemorrhage. Trauma was suspected in these cases.

**Figure 4:**
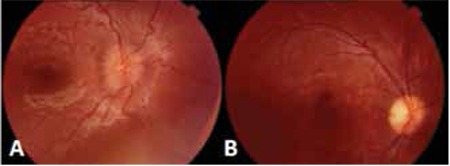
Optic disc signs in patients with acute leukemia. A. Papillary edema at optic disc. B. Atrophy at optic disc

Optic disc manifestations were observed in 9 patients (1 FAA and 8 FDF). The patient with involvement at admission had Philadelphia chromosome positive T-cell ALL. He had papilledema with optic nerve and CNS involvement at the time of diagnosis (Figure 4). The Non-Hodgkin Lymphoma-Berlin-Frankfurt-Munster-1990 (NHL-BFM 90) treatment protocol was initiated, along with cranial radiation therapy. Ophthalmic examination was normal 3 months after the initiation of ALL treatment. He has been in remission for the last 4 years.

Cranial nerve manifestations were noted in 7 patients (2 FAA and 5 FDF). Both patients with primary involvement had Horner’s syndrome at the time diagnosis; one of the patients was an 8-year-old male with AML-M4 with inversion of chromosome 16. After treatment, he has been well for the last 10 years without any ocular problems. The other patient was a 3-year-old male with B-cell ALL. This patient has been in remission for the last 5 years and his ophthalmic examination was normal at the last visit.

**Figure 5:**
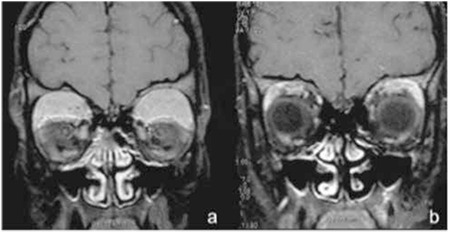
Orbital magnetic resonance images of patient with orbital granulocytic sarcoma before (a) and after (b) treatment

Orbital manifestations were recorded in 6 patients (4 FAA and 2 FDF). All orbital involvements at admission diagnosed with orbital granulocytic sarcoma were patients with AML (Figure 5). Three of them also had retinal involvement. All granulocytic sarcomas disappeared within 10 days of AML treatment.

In total, 2 patients, one with ALL-L1 and the other with AML-M5 had choroidal manifestations; both had involvement at admission and serous retinal detachment due to choroidal infiltration. They both returned to normal with leukemia treatment.

Two patients with ALL developed corneal pathology during follow-up that was related to toxicity of chemotherapeutics. One patient had evidence of subcapsular cataract as a secondary manifestation, which was also related to chemotherapy.

During bone marrow relapse, 5 patients had ocular manifestations; retinal manifestations during follow-up were observed in 3 of them (2 intraretinal hemorrhage and 1 retinitis) and cranial nerve findings were noted during follow-up in 2 patients.

## DISCUSSION

As the life expectancy of children with leukemia has increased due to advances in diagnosis and treatment methods, observation of the complications associated with leukemia has also increased. The limited studies on ophthalmologic signs in acute leukemia showed that the use of modern treatments has caused an increase in secondary ophthalmologic manifestations.^6^ Ophthalmologic manifestations in pediatric leukemia patients may occur either directly due to leukemia or as a result of problems that occur during the course of disease. Although our study was retrospective in design, the ophthalmologic manifestations of all patients, both at the time of diagnosis and as needed during the course of illness, were observed and followed by experienced ophthalmologists at the same center; therefore, we think this study is comparable in nature to a prospective study. Although age and gender distribution in the study population was normal, the percentage of patients with AML (approximately 30%) was higher than in previously reported (15-20%) studies,^7^ which might have been because patients referred to our hospital were mostly children with AML.

Russo et al.^8^ studied ophthalmologic manifestations in 180 children with acute leukemia and observed that ocular manifestations occurred with a higher frequency in AML patients than in those with ALL. In addition, bone marrow relapse developed more frequently in the patients with specific ocular manifestations than in the patients with non-specific eye lesions or non-ophthalmologic manifestations. Furthermore, CNS and bone marrow relapse occurred more frequently in the patients with specific ocular lesions, resulting in a decrease in mean duration of survival. In the present study ocular involvement occurred more frequently in patients with AML compared to those with ALL, but the difference was not significant (p=0.053). There was no correlation between ophthalmologic manifestations and the frequency of relapse, morphologic or immunophenotype, or gender distribution in the present study. Our study includes a considerable number of patients with acute leukemia; however, inconsistent results compared to the study mentioned above indicates the need for larger/multi-centric studies.

Many studies have examined the effect of age on prognosis in acute leukemia.^9^ In the present study, the occurrence of ophthalmologic manifestations increased with age in children with leukemia. Age is a strong prognostic factor in ALL, therefore our finding that the ALL patients with ophthalmologic findings were older than those without ophthalmologic findings supports this notion. However, in AML the age is not a strong prognostic factor.^7^ Our study suggests that children with AML, especially those in the older age group, should be considered for ophthalmologic findings. Nevertheless, this finding should also be confirmed with further studies.

With the advent of modern treatment regimens, some prognostic factors that were once important are becoming less significant. As such, identification of novel risk factors that can be used to inform patient treatment and follow-up are needed. In 1976 Ridgway et al.^10^ reported that among 657 children admitted with acute leukemia, 9% had ocular signs, the most common of which was retinal hemorrhage. In addition, 29 patients with leukemic involvement in the eyes mostly had bone marrow relapse, of which 27 patients had meningeal involvement based on evaluation of cerebrospinal fluid. None of these patients were treated with CNS prophylaxis, because the chemotherapy protocols did not include CNS prophylaxis at that time. In the present study 7 (17%) patients had CNS involvement with ocular manifestations (3 optic disc, 2 orbital, and 2 cranial nerve involvement) at the time of diagnosis. Only 1 patient who had CNS relapse also had orbital relapse. These findings suggest that including CNS prophylaxis in leukemia protocols prevents ocular involvement; however, larger scale studies are needed to confirm this conclusion.

In a recent report, Curto et al.^11^ revealed that 32 of 38 patients with leukemic ocular involvement remitted, but that 6 of those patients had ocular relapse; therefore, more aggressive treatment was suggested in patients with ocular involvement, which could improve not only survival, but visual function as well. According to the literature, CNS and bone marrow relapse are more common in ALL patients with ocular signs, and result in a decrease in the duration of survival.^6,12^ Therefore, the presence of ophthalmologic manifestations was thought to be a marker of poor prognosis. However, the present study’s findings do not support this notion. In the present study there was no difference in survival between the ALL patients with and without ocular signs, and survival and relapse rates were similar overall in children who had ophthalmological manifestations at admission compared to those without ophthalmological findings.

Several reports indicated that the most common ocular signs were retinal findings, of which the most common was retinal hemorrhage.^12,13,14,15^ Lower platelet counts and higher leukocyte counts were found in the acute leukemia patients with intraretinal hemorrhage. The researchers suggested that ocular signs in acute leukemia might be associated with the leukocyte and platelet counts. In the present study retinal involvement was the most common ophthalmic manifestation, consistent with these reports. However, we found no correlation between either retinal manifestations at admission or during follow-up and hematologic parameters, especially the leukocyte and platelet counts. Similarly, Ergur et al.^16^ studied 42 children with acute leukemia and reported that 24 had ocular manifestations, but that ophthalmic manifestations and hematological parameters were not correlated.

## CONCLUSION

In the present study, ophthalmic manifestations at admission or during follow-up differed according to gender and type of leukemia, and increased in frequency with age at admission. Ophthalmic manifestations were observed more frequently in the patients with AML than in those with ALL, but the difference was not significant. Despite various recommendations regarding the treatment of patients with primary ocular involvement, definitive information is still lacking. As this study did not directly demonstrate that ophthalmic manifestations negatively affect prognosis, alterations to current treatment protocols are not recommended. Multi-centric studies with larger patient groups are needed to further elucidate this issue.

## Ethics

Ethics Committee Approval: The present study was performed in accordance with the ethical standards set forth in the 1964 version of the Declaration of Helsinki, and the Başkent University Ethics Committee approved the study protocol, Informed Consent: “Informed Consent Forms” were approved by all patients’ parents.

Peer-review: Externally peer-reviewed.

## Figures and Tables

**Table 1:**
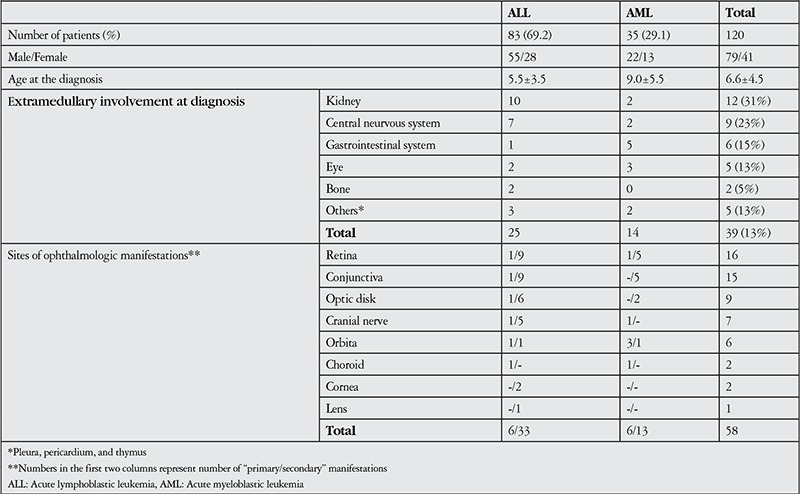
Demographic and organ involvement data of patients with leukemia
